# A2B Blood Group Without Anti-A1 Lectin Antibodies in a Child With an Enzymopathy Hemolytic Disease

**DOI:** 10.7759/cureus.20815

**Published:** 2021-12-29

**Authors:** Fadi Busaleh, Dunya Bu-Izran, Zainab Alhajji, Rawya Qahtan, Abdulatif Alnaaim, Haider Alnofaily, Salah Almohammed

**Affiliations:** 1 Pediatrics, Maternity and Children Hospital (MCH), Al-Ahsa, SAU; 2 Medicine, King Faisal University, Al Hofuf, SAU; 3 Pediatric Medicine, Maternity and Children Hospital (MCH), Al-Ahsa, SAU; 4 Family Medicine, Ministry of Health, Al-Hasa, SAU; 5 Medicine, Medical School of Olsztyn, Olsztyn, POL; 6 Pediatrics, Ministry of Health Holdings, Al Hufof, SAU

**Keywords:** packed red blood cell transfusion, non-immune hemolytic anemia, abo blood-group system, blood group, anemia

## Abstract

Generally, within the ABO blood group system, the AB group is subdivided into two subtypes, A_1_B and A_2_B, with the A_2_B subtype considered to be the rarest and the A_1_B subtype the most common. Given that the A_2_B subtype is the rarest one, its presence is associated with many challenges. In this report, we present the case of a child with a chronic hemolytic disease with the A_2_B blood group but without anti-A1 lectin antibodies, as well as the challenges encountered.

## Introduction

Packed red blood cell (PRBC) transfusion is a modality used in the management of severe anemia to restore the body’s oxygen-carrying capacity to meet the body’s metabolic demands [1]. In general, the compatibility of a transfusion is determined by blood group systems. These blood group systems are numerous, reaching up to 36 systems, among which there are a major system and a minor system [1]. The ABO blood group system is a primary system that determines the initial compatibility in blood transfusion medicine. It consists of four main groups and other minor groups. In this report, we present the case of a child with an A_2_B blood group and acute hemolytic anemia, as well as the challenges encountered [2-3].

## Case presentation

A two-year-old boy was admitted to the Maternity and Children Hospital, Al-Ahsa, Saudi Arabia, with acute hemolytic anemia secondary to glucose-6-phosphate dehydrogenase (G6PD) deficiency. The patient presented with a progressive pallor and red-colored urine associated with a decrease in activity for one day. This episode was preceded by the ingestion of fava beans two days prior to presentation. On examination, the patient was pale and lethargic but not jaundiced. He was also tachycardic (145 bpm) and had a mid-systolic murmur at the upper-left sternal border (grade 3) with no radiation or thrill and with an unremarkable systemic examination. Initial laboratory investigations revealed a decrease in the levels of hemoglobin (6.0 g/dL) with hemoglobinuria (Table 1). Therefore, the patient required a red blood cell transfusion because of his symptomatic anemia and critical levels of hemoglobin. Crossmatching prior to transfusion revealed that the child has an A_2_B blood group with positive RhD. Therefore, he was transfused with 15 mL/kg compatible type B RhD+ blood group PRBCs to avoid developing anti-A antibodies. After the transfusion, the patient stabilized hemodynamically and his hemoglobin levels increased back to 8 g/dL. His urine also became clear within a day of admission, indicating the cessation of hemolysis. Therefore, he was discharged on the second day on 1 mg of folic acid once daily for a month, to be followed up by a pediatric hematologist.

**Table 1:** Laboratory findings observed in the patient G6PD: glucose-6-phosphate dehydrogenase; HbA1: hemoglobin A1; HbA2: hemoglobin A2

Complete Blood Count		
Test	Result	Reference range
White blood cell count	18.6 × 10^3^ mg/dL	3–14 × 10^3^ mg/dL
Hemoglobin	6.0 g/dL	11.1–12.6 g/dL
Platelets	316 × 10^3^	150–350 × 10^3^
Mean corpuscular volume	82 fL	77–87 fL
Reticulocyte count	7.94%	0.5%–1.5%
Blood Chemistry Tests		
Test	Result	Reference range
Serum sodium	135 mmol/L	133–152 mmol/L
Serum potassium	4.8 mmol/L	4.1–5.3 mmol/L
Serum calcium	2.02 mmol/L	2.12–2.52 mmol/L
Serum magnesium	0.95 mmol/L	0.74–0.99 mmol/L
Serum chloride	98 mmol/L	98–115 mmol/L
Serum phosphate	1.05 mmol/L	0.8–1.5 mmol/L
Blood glucose	91 mg/dL	74–106 mg/dL
Blood urea nitrogen	4.87 mmol/L	1.70–8.30 mmol/L
Creatinine	23.46 μmol/L	49–115 μmol/L
Total bilirubin	1.4 mg/dL	0.2–1.0 mg/dL
Direct bilirubin	0.34 mg/dL	0.00–02 mg/dL
Alkaline phosphatase	229 units/L	46–116 units/L
Hematological work up		
Test	Result	Reference range
ABO blood group	A2B	A,B and O
RhD blood group	Positive	Positive or Negative
Direct Combs test	Negative	Negative
G6PD enzyme activity	Deficient	Normal
HbA1	97.3%	97.8%–97.8%
HbA2	2.7%	2.2%–3.2%
Hemoglobin H preparation	Negative	Negative
Anti-A antibodies	Negative	Negative
Anti-B antibodies	Negative	Negative
Urine analysis		
Red blood cells	0	<5 RBC/hpf
White blood cells	0	<5 WBC/hpf
Hemoglobinuria	+2	Negative

## Discussion

Since the discovery of the ABO blood group system early in the 20th century by Karl Landsteiner, its antigen compatibility has been considered central for decisions regarding transfusion adaptability. In general, according to the oligosaccharide antigens present on the cell membrane of red blood cells, the ABO blood group system is divided into four main groups: A, B, AB, and O. Two other subgroups are also rarely observed: A_2_ and A_2_B. The rarest of these subtypes is A_2_B, which is present in only 1% of the world’s population [3]. Moreover, around one-third of A_2_B blood group carriers have spontaneously formed anti-A antibodies [4]. However, even though our patient is an A2B blood group carrier, he did not have anti-A antibodies.

In blood transfusion medicine, the AB blood group is considered a universal recipient, whereas the O blood group is considered a universal donor [5]. According to Saboor et al., transfusion between individuals with an identical blood group is the best choice. However, because of the rarity of the A_2_B blood group and the difficulty of finding a compatible match, the O blood group can be used instead [3]. Since our patient had a blood group mimicking the AB blood group but had no anti-A or anti-B antibodies, this allowed us to use either type A or B blood for transfusion. However, we chose type B blood because he may develop a risk of forming anti-A antibodies (anti-A1 lectin antibodies) later on [4].

Generally, hemolytic anemia secondary to a G6PD deficiency is a benign disease that can be controlled by avoiding triggering factors, such as oxidative stress. Special dietary habits should be followed, including the avoidance of fava beans and their products, as they are considered the main triggering factor for hemolysis in G6PD-deficient patients. It is also important to avoid oxidative agents, such as sulfa drugs, or some types of toilet deodorants (e.g., naphthalene balls), which may induce hemolysis [6]. Since our patient was G6PD deficient, we extended our blood group antigen matching to include minor blood groups. This was done to avoid subsequent alloimmunization with recurrent PRBC transfusions [7].

## Conclusions

Blood transfusion is a life-saving procedure with a high risk of complications. Its compatibility is determined by multiple antigens, most importantly the ABO blood group system, with its four main types. Therefore, it is important to have better knowledge of the compatibility and complications of transfusion that may be encountered, especially for patients with chronic hemolytic diseases who require recurring transfusions.
